# Inhibiting PAD2 enhances the anti-tumor effect of docetaxel in tamoxifen-resistant breast cancer cells

**DOI:** 10.1186/s13046-019-1404-8

**Published:** 2019-10-10

**Authors:** Fujun Li, Lixia Miao, Teng Xue, Hao Qin, Santanu Mondal, Paul R. Thompson, Scott A. Coonrod, Xiaoqiu Liu, Xuesen Zhang

**Affiliations:** 1 State Key Laboratory of Reproductive Medicine, Nanjing Medical University, Nanjing, 211166 China; 2 Department of Obstetrics and Gynecology, The Affiliated Jiangning Hospital of Nanjing Medical University, Nanjing, 211100 China; 3 Department of Biochemistry and Molecular Pharmacology, University of Massachusetts Medical School, Worcester, MA 01655 USA; 4 Baker Institute for Animal Health, College of Veterinary Medicine, Cornell University, New York, 14853 USA; 5 Key Laboratory of Pathogen Biology of Jiangsu Province, Department of Microbiology, Nanjing Medical University, Nanjing, 211166 China

**Keywords:** Breast cancer, Tamoxifen-resistance, PAD2, Docetaxel

## Abstract

**Background:**

Tamoxifen resistance presents a huge clinical challenge for breast cancer patients. An understanding of the mechanisms of tamoxifen resistance can guide development of efficient therapies to prevent drug resistance.

**Methods:**

We first tested whether peptidylarginine deiminase 2 (PAD2) may be involved in tamoxifen-resistance in breast cancer cells. The effect of depleting or inhibiting PAD2 in tamoxifen-resistant MCF-7 (MCF7/TamR) cells was evaluated both in vitro and in vivo. We then investigated the potential of Cl-amidine, a PAD inhibitor, to be used in combination with tamoxifen or docetaxel, and further explored the mechanism of the synergistic and effective drug regimen of PADs inhibitor and docetaxel on tamoxifen-resistant breast cancer cells.

**Results:**

We report that PAD2 is dramatically upregulated in tamoxifen-resistant breast cancer. Depletion of PAD2 in MCF7/TamR cells facilitated the sensitivity of MCF7/TamR cells to tamoxifen. Moreover, miRNA-125b-5p negatively regulated PAD2 expression in MCF7/TamR cells, therefore overexpression of miR-125b-5p also increased the cell sensitivity to tamoxifen. Furthermore, inhibiting PAD2 with Cl-amidine not only partially restored the sensitivity of MCF7/TamR cells to tamoxifen, but also more efficiently enhanced the efficacy of docetaxel on MCF7/TamR cells with lower doses of Cl-amidine and docetaxel both in vivo and in vivo. We then showed that combination treatment with Cl-amidine and docetaxel enhanced p53 nuclear accumulation, which synergistically induced cell cycle arrest and apoptosis. Meanwhile, p53 activation in the combination treatment also accelerated autophagy processes by synergistically decreasing the activation of Akt/mTOR signaling, thus enhancing the inhibition of proliferation.

**Conclusion:**

Our results suggest that PAD2 functions as an important new biomarker for tamoxifen-resistant breast cancers and that inhibiting PAD2 combined with docetaxel may offer a new approach to treatment of tamoxifen-resistant breast cancers.

## Background

Despite major advances in the development of antineoplastic agents, breast cancer remains as one of the most prevalent malignant tumors and the first leading causes of cancer morbidity and mortality in women worldwide [1]. About 70% of breast cancers are estrogen receptor (ER) positive. Tamoxifen, as one of the most prescribed ER antagonists for first line adjuvant endocrine therapy, has significant efficacy for ER-positive breast cancer, and has been shown to substantially reduce recurrence and mortality rate in ER-positive breast cancer patients [2]. Unfortunately, with 5 years of tamoxifen therapy, most initially responsive patients experience a recurrence, and the tumors are eventually resistant to tamoxifen [3, 4]. Therefore, the search for the mechanisms responsible for endocrine resistance and effective treatment therapies for these breast cancers continues, which has increased the popularity of combined therapies of two or more cancer drugs [5].

A recent study showed that tamoxifen-resistant breast cancer cells are resistant to DNA-damaging chemotherapies, including cisplatin and adriamycin, but not to paclitaxel, suggesting that taxane-based chemotherapy may be superior to DNA-damaging drugs when choosing chemotherapy for tamoxifen-resistant breast cancer patients [6]. Docetaxel (formerly called taxotere) is a second-generation taxane which is widely used in cancer therapy, either as a monotherapy or as a combination therapy across a variety of tumor types [7]. Currently, docetaxel is still the first-line chemotherapy in breast cancer and constitutes one of the most effective classes of chemotherapeutics for prolonged survival in advanced disease [8, 9]. Clinical studies also showed that combination of docetaxel with other cancer drugs yields good results [5]. However, cumulative systemic toxicity after prolonged and high-dose therapy was found to be associated with safety issues among clinical trial patients including hematological issues, asthenia, cutaneous reactions, and neurosensory reactions [5, 10]. Clearly, it is of high clinical significance to enhance the efficacy of docetaxel using lower doses in a less toxic manner and to reduce its side effects. Therefore, therapeutic strategies that can either increase the effects of chemotherapeutics or decrease the dosage are urgently needed for the treatment of tamoxifen-resistant breast cancers.

Peptidylarginine deiminases (PADs) are a family of calcium dependent enzymes that convert arginine into citrulline in substrate proteins. Accumulating studies on the PADs have gathered increased attention due to their emerging roles in various human and animal cancers [11, 12]. For example, we recently demonstrated that PAD1 was upregulated in triple negative breast cancer and regulated cancer cell metastasis by targeting MEK1 in ERK signaling pathway [13]. Other studies showed that PAD2 and PAD4 were detected in a wide range of human malignant cancers and suggested a link with cancer progression [11, 14–18]. During the transition from benign mammary epithelium to malignant breast carcinomas, PAD2 expression was shown to be dramatically upregulated [17]. A recent study also showed that PAD2 targets RNA polymerase II to facilitate gene expression and cell proliferation in breast cancer cells [19]. In line with these observations, PAD2 overexpression in breast cancer and other cancers correlates with poor prognosis [20–22]. Given the upregulated expression level of PAD2 in breast cancers, depletion or inhibiting PAD2 should decrease tumor growth. In fact, McElwee and colleagues demonstrated that a pan-PAD inhibitor, Cl-amidine, strongly suppressed breast cancer cell growth by altering the expression of both cell cycle and tumor promoting genes [17]. Meanwhile, Wang and colleagues developed a novel PAD inhibitor, YW3–56, also inhibits cancerous growth by perturbing autophagy [23]. These studies suggest that overexpression of PAD2 plays a pivotal role in regulating tumor progression, which may open the possibility that specific inhibition of PAD2 activity may represent a suitable drug target for new breast cancer therapies.

However, nothing is known about whether PAD2 is involved in endocrine resistance in breast cancers. Our goal here was to formally test this hypothesis and then investigate the effects of PAD2 inhibition in combination with tamoxifen or docetaxel in cell culture, and preclinical in vivo models of breast cancer. Our study is anticipated to provide a novel therapeutic approach to improve clinical practice in treatment of tamoxifen-resistant breast cancer with enhanced drug efficacy and reduced side effects.

## Methods

### Cell culture

Tamoxifen-sensitive (TamS) and resistant (TamR) MCF-7 cells were a gift from Dr. Joshua LaBaer at Biodesign Institute. HEK293 and TamS cells were grown in Dulbecco’s Modified Eagle Medium (DMEM) supplemented with 10% fetal bovine serum (FBS) and 1% Penicillin Streptomycin. TamR cells were grown in the same media supplemented with 1 μM tamoxifen (Sigma-Aldrich, USA). All cells were maintained at 37 °C in a humidified 5% CO_2_ atmosphere. PAD2-depleted TamR/MCF-7 cells were generated by transduction with Mission Lentiviral Transduction Particles containing a short hairpin RNA (shRNA) construct targeting the human PAD2 coding sequence (Sigma SHCLND-NM_007365). In the control group, cells were transduced with a non-targeting shRNA lentiviral construct (Sigma SHC002V). Cells were selected by medium containing 1 μg/mL puromycin (Sigma-Aldrich). For generating miR-125b-5p overexpression TamR/MCF7 cells, retroviral particles containing genomic DNA fragment (hg38_wgRna_hsa-mir-125b-1 range = chr11:122099595–122,100,001) cloned into pQXCIP construct were generated to infect TamR/MCF7 cells. In the control group, TamR/MCF7 cells were transduced with an empty pQXCIP construct. Cells were selected by medium containing 1 μg/mL puromycin (Sigma-Aldrich). Where indicated, Cl-amidine or docetaxel (Sigma-Aldrich) was diluted in cell culture medium at the indicated concentration.

### Cell proliferation assay and colony formation assay

Cells were seeded into 96-well plates (5000 cells/well), incubated overnight and then treated with or without Tamoxifen for 1, 2, 3, 4 and 5 days. 10 μL of cell counting kit-8 (CCK-8) reagent (Yeasen, Shanghai, China) was added to each well, and plates were incubated for 4 h at 37 °C in accordance with the CCK-8 kit protocol. Optical density (OD) values were measured at 450 nm using a plate reader (Thermo Scientific Multiskan GO, Finland). For colony formation assay, a single-cell suspension was seeded into 6-well plates and grown for 4 days in the presence of 7 μM tamoxifen, fixed with 4% paraformaldehyde, and stained with crystal violet for subsequent colony counting analysis.

### Apoptosis evaluation by flow cytometry and TUNEL assays

Apoptotic cells were detected using the Annexin V-fluorescein isothiocyanate (FITC) Apoptosis Detection Kit (Yeasen, Shanghai, China). Briefly, following treatment of cells, cells were washed and then cell pellets were re-suspended in ice-cold binding buffer. Subsequently, 5 μL Annexin V-FITC solution and 5 μL dissolved propidium iodide (PI) were added to the cell suspension. After gentle mixing, samples were incubated for 10 min in the dark at room temperature. A FACScan flow cytometer was applied to quantify cellular apoptosis. For terminal deoxynucleotidyltransferase mediated dUTP-biotin nick end labeling (TUNEL), cells were grown on glass slides in 12-well plates, followed by fixation with 4% paraformaldehyde. TUNEL staining was performed with a TUNEL Apoptosis Detection Kit (FITC) (Yeasen, Shanghai, China) according to the manufacturer’s instructions. Following TUNEL staining, cells were washed and then blocked with 4% bovine serum albumin for 5 min at room temperature. Nuclei were visualized by DNA staining with Hoechst stain (1 μg/mL). The images were captured using a Carl Zeiss (Germany). TUNEL positive signal was counted from randomly selected fields.

### Quantitative real-time PCR

Total RNA was extracted using TRIzol (Invitrogen). RNA quality and quantity were quantified with Nanodrop 2000 spectrophotometer (Thermo Scientific). 500 ng total RNA was reversely transcribed into cDNA using SuperScript™ III Reverse Transcriptase (Invitrogen). Quantitative real-time PCR was performed using the Power SYBR Green PCR Master Mix (Applied Biosystems) with gene-specific primers. The primers used were summarized in Additional file 2: Table S1. The relative fold expressions were calculated using relative standard curve method (2^−ΔΔCt^). For the analysis of cell apoptosis, cell cycle and autophagy gene expression, cDNA was analyzed using human Qiagen RT^2^ Profiler PCR Cell Apoptosis Array (PAHS-012Z), Cell Cycle Array (PAHS-020Z), and Autophagy Array (PAHS-084Z), separately. Data were normalized using multiple housekeeping genes and analyzed by comparing 2^−ΔΔCt^ of the normalized sample.

### Western blot analysis

Radioimmunoprecipitation assay (RIPA) buffer containing protease inhibitors was used to extract total proteins, and the lysates were boiled for 5 min before subjected to 10% SDS-PAGE. The proteins were then transferred to PVDF membranes. The membranes were blocked and incubated with the following primary antibodies overnight at 4 °C: PAD1, PAD4 (Sigma-Aldrich); PAD2 (Proteintech); Bcl-2, Bak, Bad, LC3B, GSK3β, caspase 3, cleaved caspase 3, p-Rps-6, Rps-6, p-Akt, Akt (Cell Signaling Technology, USA), p53 (Bioworld Technology, China), and PAD3, GAPDH (Santa Cruz Biotechnology, USA). The membranes were washed and then incubated with HRP-conjugated secondary antibodies. The signals were visualized using an Enhanced Chemiluminescence Detection Kit (Pierce Biotechnology, USA).

### Immunofluorescence staining

Cells were grown on glass slides in 12-well plates, then fixed with 4% paraformaldehyde and permeabilized with 0.1% Triton X-100. After blocking, cells were incubated with primary antibodies against LC3B. Then Fluor 555-conjugated secondary antibody (Invitrogen) was employed to detect fluorescence. The nuclei were stained with DAPI (Vector Laboratories, Cambridgeshire, UK). Representative images were collected with LSM 510 laser scanning confocal microscope (Carl Zeiss).

### Nuclear and cytoplasmic extract preparations

Cells were washed twice with cold PBS and then lysed in cold cell lysis buffer (1 M Tris-HCl, pH = 7.9; 1 M KCl, 10% NP40, 1x proteinase inhibitors) for 60 min on ice. The lysates were then centrifuged and supernatants were collected as cytoplasmic fraction. The pellets were washed and then lysed in cold lysis buffer (1 M Tris-HCl, pH = 7.9; 0.5 M EDTA, 10%SDS, 1x proteinase inhibitors). The supernatants were collected as nuclear fraction.

### Immunoprecipitation assay

Flag-tagged PAD2 in pcDNA3.1(+) and HA-tagged-Ub were transfected into HEK293 cells using FuGENE 6 (Roche). Cells were collected and lysed 40 h post transfection and the whole cell lysates were immunoprecipitated with anti-p53 antibody. Immunoprecipitates were then washed and analyzed by western blot using anti-HA antibody (Bioworld Technology, China). GAPDH was used as a control as indicated.

### Xenograft tumor model in nude mice

Female BALB/c nude mice (6-week-old) were purchased from the Shanghai Laboratory Animal Center (Chinese Academy of Sciences, Shanghai, China) and maintained in a special pathogen-free environment. All procedures were reviewed and approved by the Institutional Animal Care and Use Committee of Nanjing Medical University. Cells (1 × 10^7) were injected subcutaneously into the left upper flank of mice. Tumor diameters are measured with digital calipers, and the tumor volume in mm^3^ was calculated by the following formula: Volume = 0.5 x (Width)^2^ x Length (*n* = 3). Alternatively, the tumor weight was recorded (n = 3). For the experiment examining the effect of PAD2 inhibitor and docetaxel on tumor growth, TamR/MCF-7 cells were injected subcutaneously into 24 female nude mice. The tumors were grown for 2 weeks. Mice were randomly assigned into 4 groups (*n* = 6) and administered intra-peritoneal injections of either Cl-amidine (20 mg/kg/day) alone, docetaxel (10 mg/kg/day) alone, or a combination of Cl-amidine and docetaxel every 3 days. Treatment continued for 3 weeks and the mice were then sacrificed under anesthesia. The tumor weight was recorded. PBS was used as injection control.

### Statistical analysis

All experiments were independently repeated at least three times. Data are presented as mean ± SD. Statistical evaluation for data analysis was determined by Student’s t-test with *indicating means significantly different (*P* < 0.05) from control.

## Results

### PAD2 expression is highly upregulated in tamoxifen-resistant breast cancer, and depletion of PAD2 facilitates the sensitivity of MCF7/TamR cells to tamoxifen

**Fig. 1:**
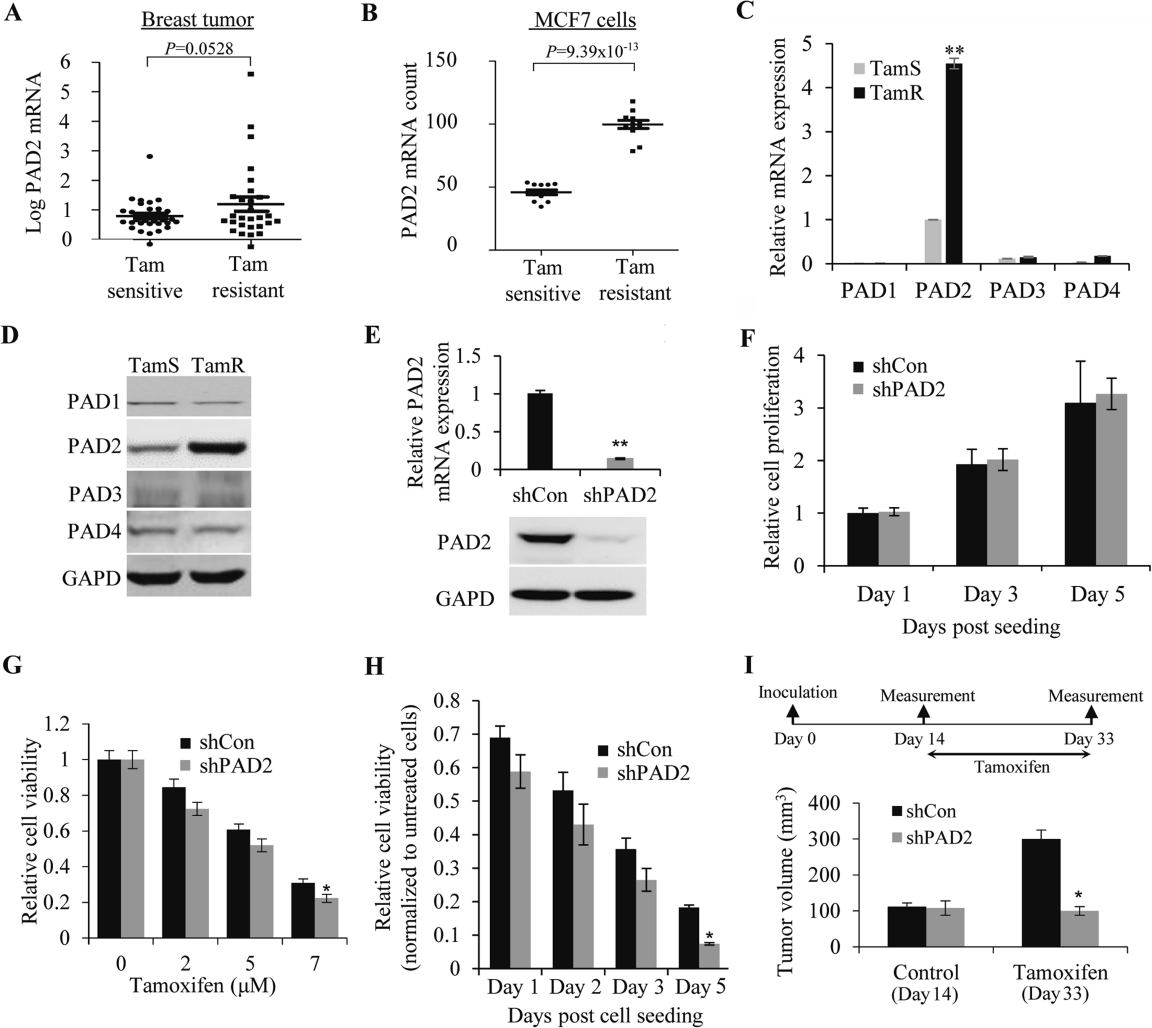
PAD2 expression is highly upregulated in tamoxifen-resistant breast cancer, and depletion of PAD2 facilitates the sensitivity of MCF7/TamR cells to tamoxifen. **a** Analysis of PAD2 mRNA levels in clinical tumor tissue microarray during tamoxifen therapy using the publicly GEO dataset GDS806/11785. **b** Endogenous PAD2 mRNA levels in tamoxifen-resistant breast cancer cell line MCF7 (MCF7/TamR) were compared to tamoxifen-sensitive controls (MCF7/TamS). GAPDH served as controls. **c**, **d** Endogenous PAD1–4 mRNA and protein levels in MCF7/TamR cells were determined by qRT-PCR (**c**) and Western Blot (**d**). GAPDH served as loading control. **e** Stable PAD2 knockdown efficiency was confirmed by qRT-PCR and immunoblot. GAPDH served as loading control. **f** MCF7/TamR cell proliferation was not affected upon PAD2 depletion by CCK8 assay, compared to the empty vector (shCon) control cells. **g** Cell proliferation was inhibited in PAD2 knockdown MCF7/TamR cells by CCK8 assay, in the presence of 7 μM tamoxifen, compared to the empty vector (shCon) control cells. **h** 7 μM tamoxifen treatment on TamR/MCF7 cells showed a time-dependent inhibition of cell proliferation. *P < 0.05. **i** The mice bearing PAD2 knockdown cells exhibited smaller tumors than the mice with shRNA control cells post tamoxifen treatment (*n* = 3/group). The volume of tumors at indicated time after cell implantation was quantified, and the average volume was plotted. *P < 0.05

To determine the clinical significance of PAD2 in tamoxifen-resistant breast tumors, we first examined PAD2 mRNA level in clinical tumor tissue microarray during tamoxifen therapy using the publicly GEO dataset GDS806/11785 (https://www.ncbi.nlm.nih.gov/geoprofiles) [24]. As shown in Fig. 1a, PAD2 transcript levels were elevated in breast tumor tissues from breast cancer recurrence group compared to those patients with disease free (tamoxifen sensitive) during tamoxifen therapy, though *P* value was more than 0.05 (*P* = 0.0528). Further analysis of PAD2 expression in tamoxifen-resistant breast cancer cell line MCF7 (MCF7/TamR) subclones confirmed that PAD2 was significantly (*P* = 9.39 × 10^− 13^) upregulated compared to the tamoxifen-sensitive controls (MCF7/TamS) (Fig. 1b). Notably, PAD2 transcript was more highly expressed in MCF7/TamS cells compared to the other PADs family members (Fig. 1c), and only PAD2 was significantly upregulated in TamR/MCF7 cell (Fig. 1c and d). These results indicate that elevated PAD2 levels, but not other PADs, are associated with tamoxifen resistance in breast cancer.

To further explore the role of PAD2 in the process of tamoxifen resistance, we then stably depleted PAD2 in TamR/MCF7 cells via a lentivirus-based approach. The knockdown efficiency of PAD2 was checked via western blot analysis (Fig. 1e). We then evaluated the effect of PAD2 depletion on viability of TamR/MCF7 cells using a CCK-8 assay. Results showed that, in the absence of tamoxifen, depletion of PAD2 did not affect cell growth, compared to the control cells (Fig. 1f). However, in the presence of 2–7 μM tamoxifen, PAD2-knockdown TamR/MCF7 cells showed a significant time-dependent inhibition of cell proliferation (Fig. 1g and h). We also confirmed the in vitro phenotype of TamR/MCF7 cells under tamoxifen treatment in xenograft mouse model. The PAD2-knockdown or the control TamR/MCF7 cells were inoculated into the nude mice, separately. Two weeks later, both cell lines were able to generate similar size tumors. The mice from both groups were then administrated with 3 mg/kg/day tamoxifen for additional 19 days. The mice bearing PAD2 knockdown cells exhibited significantly smaller tumors than the mice with shRNA control cells under tamoxifen treatment (Fig. 1i). These results suggest that PAD2 depletion partially restores the sensitivity of TamR/MCF7 cells to tamoxifen, and also raised a possibility that inhibiting PAD2 might help reverse tamoxifen resistance.

### MiR-125b-5p negatively regulates PAD2 expression in MCF7/TamR cells, and overexpression of miR-125b-5p increases the sensitivity of MCF7/TamR cells to tamoxifen

**Fig. 2:**
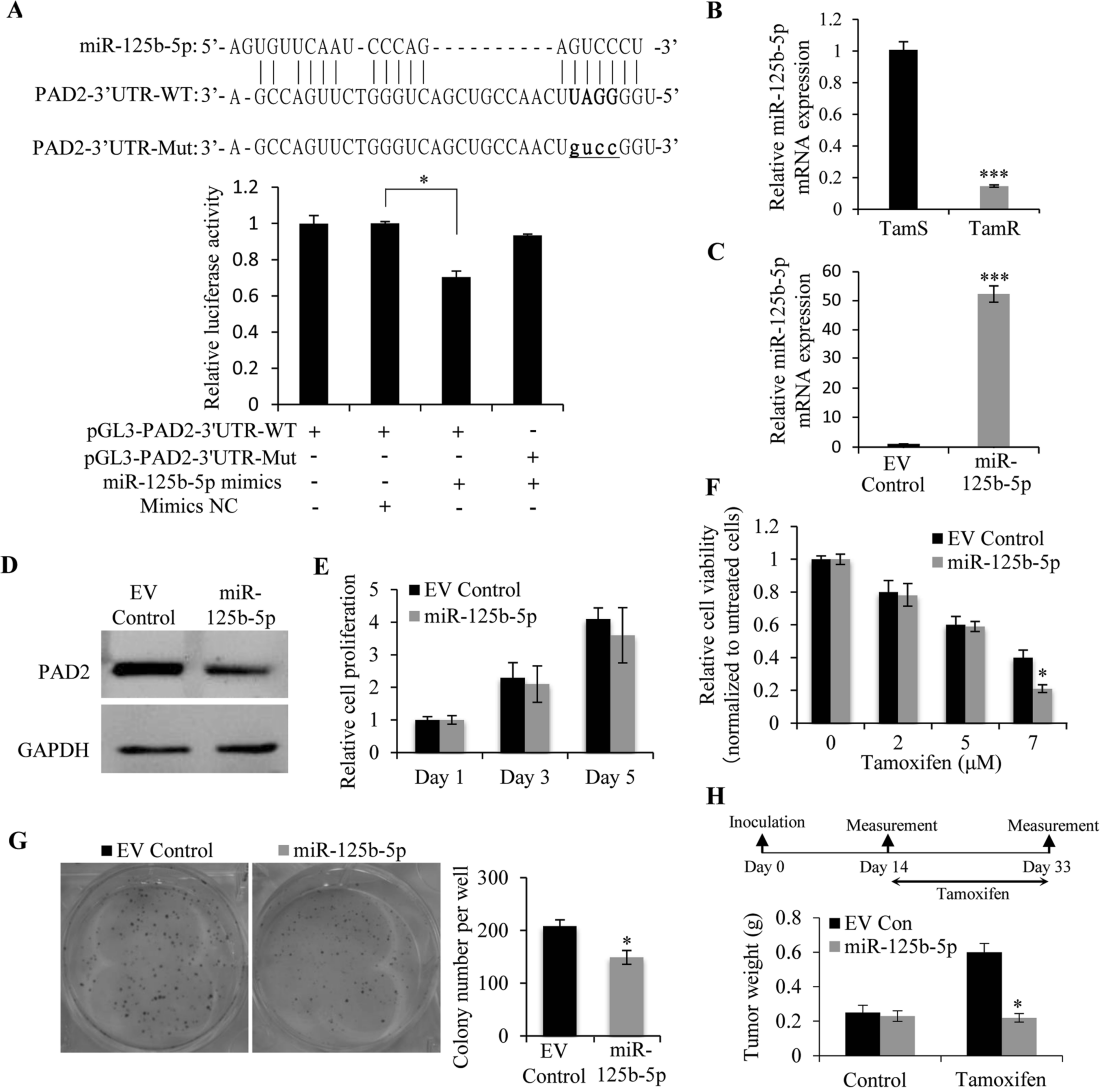
MiR-125b-5p down-regulates PAD2 by directly targeting its 3’UTR and overexpression of miR-125b-5p increases the sensitivity of MCF7/TamR cells to tamoxifen. **a** The PAD2 3’UTR contains the binding seed sequences of miR125b-5p according to online bioinformatics analysis using Bibiserv2. Mutations of the 3′-UTR of PAD2 was used to create the mutant luciferase reporter construct. The WT and Mut-PAD2 3’UTR were separately cloned into the pGL3 luciferase reporter vector, and luciferase reporter assay showed that the activity of WT-PAD2 3’UTR, but not the mutant, was repressed by miR125b-5p overexpression. *P < 0.05. **b** qRT-PCR analysis showing that the endogenous miR125b-5p mRNA level was downregulated in MCF7/TamR cells compared to that in MCF7/TamS cells. ***P < 0.001. **c** qRT-PCR analysis confirming that miR125b-5p was stably overexpressed in MCF7/TamR cells. **d** PAD2 protein expression was downregulated by western blotting in miR125b-5p overexpressed MCF7/TamR cells. **e** Cell proliferation was not affected upon in miR125b-5p overexpressed MCF7/TamR cells by CCK8 assay, compared to the empty vector (EV Control). **f** Cell proliferation was inhibited in miR125b-5p overexpressed MCF7/TamR cells, in the presence of 7 μM tamoxifen, compared to the empty vector control cells. **g** 7 μM tamoxifen treatment on miR125b-5p overexpressed MCF7/TamR cells showing that miR125b-5p overexpression decreased colony formation. The data were presented as the mean ± SD from three independent experiments (left panel). *P < 0.05. **h** The mice bearing miR125b-5p overexpressed MCF7/TamR cells exhibited smaller tumors than the mice with Empty vector control cells post tamoxifen treatment (n = 3/group). The volume of tumors at indicated time after cell implantation was quantified, and the average volume was plotted. *P < 0.05

The molecular mechanism underlying PAD2 upregulation in tamoxifen resistance is completely unknown. A recent study demonstrated that miR-125a-5p could target the 3′-untranslated region (UTR) of PAD2 to negatively regulate PAD2 expression in the process of liver metastasis of colorectal cancer [25]. To identify upstream regulators of PAD2 in MCF7/TamR cells, we first performed a bioinformatics analysis using Bibiserv2 (https://bibiserv.cebitec.uni-bielefeld.de) and found that PAD2 contains a putative binding site for miR-125b-5p (Fig. 2a), which is another miR-125 family member that has been reported to be downregulated in breast cancers [26–28]. To validate this prediction, the wild-type (WT) or mutated (Mut) 3’UTR sequences of PAD2 were cloned into pGL3 luciferase reporter vector and co-transfected with miR-125b-5p mimics or mimics controls (NC) into 293 T cells. The following luciferase reporter assay showed that miR-125b-5p mimics significantly inhibited luciferase activity of the WT-PAD2–3’UTR but not that of mutant (Fig. 2a), indicating the authentic binding between miR-125b-5p and PAD2–3’UTR. Moreover, our observation showing that miR-125b-5p transcript was dramatically downregulated in TamR/MCF7 cells (Fig. 2b) may be a causal mechanism for explaining the increased PAD2 expression in TamR/MCF7 cells. To further test this hypothesis, we stably overexpressed miR-125b-5p in TamR/MCF7 cells (Fig. 2c) and found that PAD2 expression was inhibited in miR-125b-5p overexpressed TamR/MCF7 cells (Fig. 2d). Again, we did not observe a significant difference in cell growth when miR-125b-5p was overexpressed, relative to the control cells (Fig. 2e). However, in the presence of 7 μM tamoxifen, miR-125b-5p significantly inhibited cell proliferation and colony formation abilities of TamR/MCF7 cells (Fig. 2f and g). Notably, tumor xenograft mouse model also confirmed that miR-125b-5p overexpression would partially resensitize TamR/MCF7 cells to tamoxifen (Fig. 2h). These results suggested that decreased miR-152b-5p in TamR/MCF7 cells could upregulate PAD2 expression during tamoxifen resistance in breast cancers.

### Inhibiting PAD2 combined with docetaxel synergistically inhibited MCF7/TamR cells proliferation

**Fig. 3:**
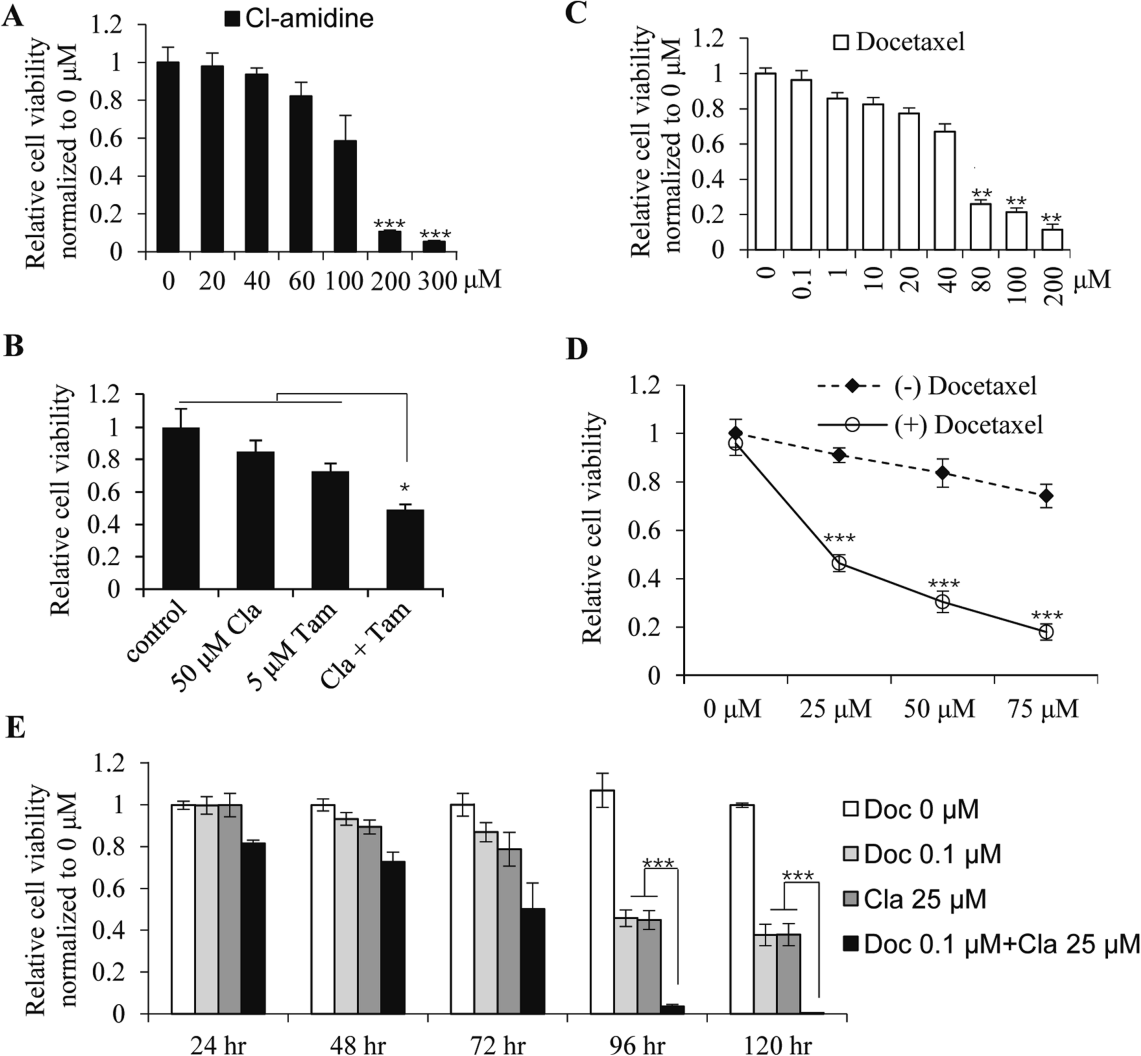
Inhibiting PAD2 re-sensitizes MCF7/TamR cells to docetaxel. **a** MCF7/TamR cells were treated with cl-amidine at the indicated concentration for 4 days. CCK8 assay showing that 200 μM cl-amidine started to inhibit MCF7/TamR cell proliferation (***P < 0.001). **b** MCF7/TamR cells were treated with cl-amidine at the indicated concentration for 4 days. CCK8 assay showing that 50 μM cl-amidine combined with 5 μM tamoxifen significantly inhibited MCF7/TamR cell growth (*P < 0.05). **c** MCF7/TamR cells were treated with docetaxel at the indicated concentration for 4 days. CCK8 assay showing that at least 80 μM of docetaxel decreased MCF7/TamR cell viability (*P < 0.05). **d** MCF7/TamR cells were treated with 0.1 μM combined with different concentration of docetaxel for 4 days. CCK8 assay showing that at least 0.1 μM docetaxel and 25 μM cl-amidine combination significantly decreased MCF7/TamR cell viability (***P < 0.001). **e** MCF7/TamR cells were treated with 0.1 μM docetaxel and 25 μM cl-amidine for 1, 2, 3, 4, and 5 days. CCK8 assay showing that this combination completely inhibited MCF7/TamR cell viability (***P < 0.001)

Given that decreased PAD2 expression resensitizes MCF7/TamR cells to tamoxifen, we determined whether inhibiting PAD2 would have the same effect. To test this hypothesis, we first treated cells with a PAD inhibitor, Cl-amidine, which elicits strong cytotoxic effects on breast cancer cells while having no observable effect on non-cancerous lines [17, 29, 30]. For these experiments, MCF7/TamR cells were incubated with increasing doses of Cl-amidine for 48 h and then evaluated for cell viability using the CCK8 assay. Cell viability was not significantly affected until the concentration of Cl-amidine reached 200 μM (Fig. 3a). Given the high concentration of Cl-amidine in the cell culture media, we next treated MCF7/TamR cells with 50 μM Cl-amidine. Results showed that 50 μM Cl-amidine treatment alone did not affect cell viability, however, 50 μM of Cl-amidine combined with 5 μM tamoxifen significantly inhibited MCF7/TamR cell growth by ~ 2-fold (Fig. 3b). These results not only confirmed our hypothesis that depletion or inhibiting PAD2 will partially restore the sensitivity of MCF7/TamR cells to tamoxifen, but also suggested that PAD2 is a good therapeutic candidate for tamoxifen resistant breast cancers.

It has been reported that combined therapies of either docetaxel or tamoxifen with other cancer drugs have yielded good results in clinical trials [5, 31]. A more recent study also showed that tamoxifen-resistant MCF7 cells are sensitive to paclitaxel, while other major chemotherapeutic drugs used in breast cancer, including cisplatin or adriamycin, are not [6]. As PAD2 appears to be a good therapeutic target for tamoxifen resistance, we determined whether inhibiting PAD2 enhances the efficacy of docetaxel on MCF7/TamR cells. To test this possibility, we first examined the cytotoxicity of docetaxel on our MCF7/TamR cells and found that 80 μM docetaxel significantly decreases cell viability (Fig. 3c). Next, docetaxel showed a synergistic effect with Cl-amidine, occurring at a low dose of 0.1 μM docetaxel (Fig. 3d). Of note, this drug regimen even decreased the concentration of Cl-amidine to as low as 25 μM, compared to 50 μM Cl-amidine in combination with tamoxifen. We then used 25 μM Cl-amidine combined with 0.1 μM docetaxel for all future experiments, based on the completely inhibiting effect on viability of MCF7/TamR cells after 5-days of treatment (Fig. 3e). Therefore, inhibiting PAD2 not only resensitized MCF7/TamR cells to tamoxifen, but also greatly enhanced the efficacy of docetaxel.

### Cl-amidine combined with docetaxel synergistically induces apoptosis, cell cycle arrest, and autophagy in MCF7/TamR cells

**Fig. 4:**
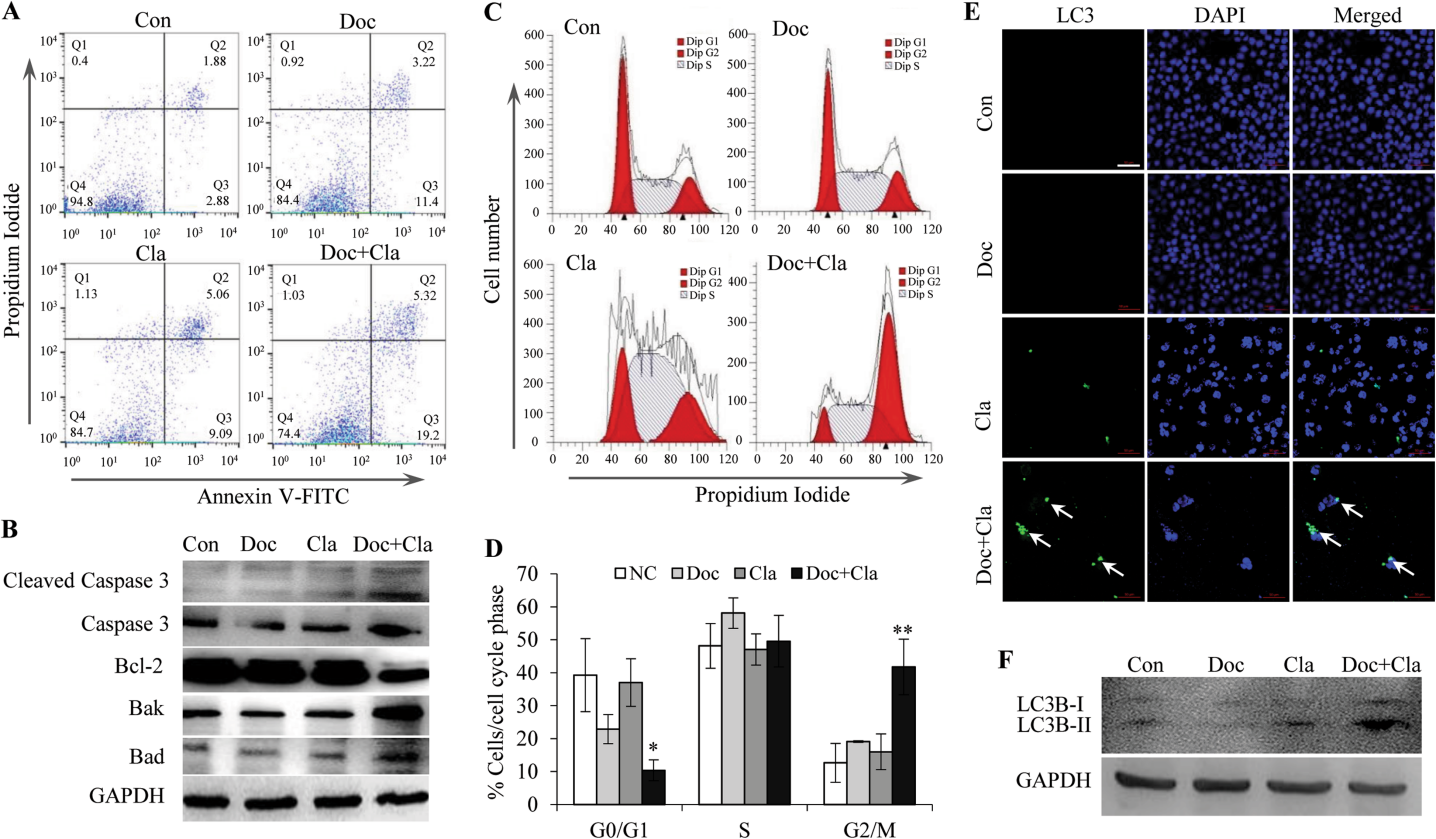
Cl-amidine combined with docetaxel synergistically induces cell apoptosis, cell cycle arrest, and autophagy in MCF7/TamR cells. **a** Flow cytometric analysis of 0.1 μM docetaxel and 25 μM cl-amidine combincation accelerated apoptosis of the MCF7/TamR cells compared to either individual treatment. **b** Western blot analysis of the activation of caspase 3, Bcl-2, Bak, and Bad in MCF7/TamR cells treated with 0.1 μM docetaxel and 25 μM cl-amidine. GAPDH was used as an internal control. **c,d** Flow cytometric analysis of 0.1 μM docetaxel and 25 μM cl-amidine combination induced cell cycle arrest in the G2/M phase compared to docetaxel or cl-amidine alone. The relative quantification was shown in (**d**). **e** Immunofluorescence staining for LC3B showing 0.1 μM docetaxel and 25 μM cl-amidine combination induced large punctate structures in MCF7/TamR cells (white arrow). Nuclei were stained with DAPI. Scale bar, 50 μm. **f** Western blot analysis of the LC3B protein greatly accumulated under the combined treatment. GAPDH served as loading control

Induction of cell death and cell cycle arrest are considered to be the main mechanisms for drug-dependent inhibition of cell growth [32]. The observed effect of Cl-amidine combined with docetaxel on cell viability suggested that the treatment might affect cell death. To follow up on the underlying mechanism of this effect, we first performed flow cytometric analysis to evaluate apoptosis. As assessed by Annexin-V staining, apoptosis was observed after exposure of MCF7/TamR cells to either docetaxel or Cl-amidine for 4 days, while the combination treatment with Cl-amidine and docetaxel significantly accelerated the apoptotic rate as compared to each individual treatment (Fig. 4a). Meanwhile, activation of caspases 3, downregulation of anti-apoptotic protein Bcl-2, and upregulation of pro-apoptotic proteins Bak and Bad in the combined treatment group confirmed the induction of apoptosis in MCF7/TamR cells (Fig. 4b). Further, cell cycle analysis with propidium iodide (PI) staining of DNA showed that Cl-amidine combined with docetaxel exhibited a much stronger cell cycle arrest in the G2/M phase compared to docetaxel or Cl-amidine alone (Fig. 4c and d), which could lead to mitotic arrest and cell growth inhibition. Autophagy can also promote cell death [33]. Thus, we tested whether the combination treatment was able to induce autophagy in MCF7/TamR cells. Using the microtubule-associated protein light chain 3B (LC3B) as a marker of autophagosomes, we observed that LC3B staining was not detected in control or docetaxel-treated cells, but presented in multiple large punctate structures after treatment with Cl-amidine and docetaxel. Though the positive signals were also observed in Cl-amidine treatment alone, the signal intensity and the number of positive LC3B puncta were less than the combined treatment (Fig. 4e). Consistent with LC3B fluorescence staining, western blotting also showed that LC3B protein greatly accumulated under the combined treatment (Fig. 4f). Interestingly, either knockdown PAD2 or overexpression of miR-125b-5p also promoted the apoptosis (Additional file 1: Figure S1), induced a stronger cell cycle arrest in the G2/M phase (Additional file 1: Figure S2), and enhanced the autophagy (Additional file 1: Figure S3) of the MCF7/TamR cells treated with 0.1 μM docetaxel. Altogether, these results suggested a promising nontoxic means by inhibiting PAD2 with Cl-amidine to enhance the efficacy of docetaxel.

### Cl-amidine combined with docetaxel alters the expression of genes associated with apoptosis, cell cycle arrest and autophagy

**Fig. 5:**
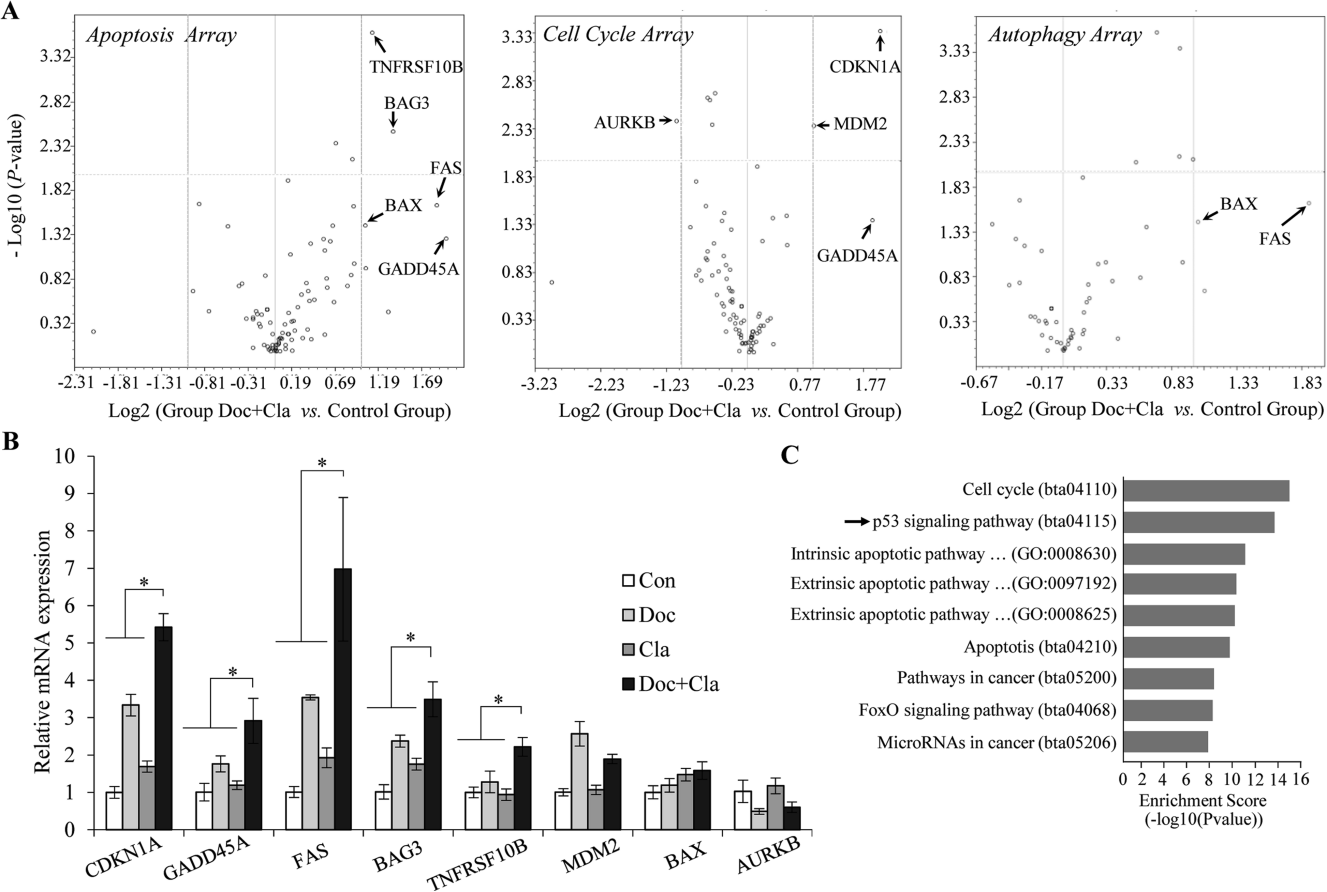
PCR array analysis of gene expression in cl-amidine combined with docetaxel treatment in MCF7/TamR cells in comparison to control cells**. a** Volcano plot human RT^2^ Profiler PCR Cell for cell apoptosis array, cell cycle array, and autophagy array. The relative expression levels for each gene depicted as log 2 (n-fold) plotted against –Log10 (*P*-value). Arrows indicating significantly upregulated or downregulated genes. **b** qRT-PCR analysis validated the expression of the genes selected from the PCR arrays in (**a**). **c** Gene ontology (GO) function analysis using DAVID Bioinformatics Resources (http://david.abcc.ncifcrf.gov/) confirming that the genes regulated by the combination treatment were enriched in p53 signaling pathway in cancers (as indicated by arrow)

The effect of combined treatment on cell growth suggested that this drug combination might affect tumor growth by altering the expression of genes involved in apoptosis, autophagy, and cell cycle progression. To test this hypothesis, mRNA from the drug-treated and control MCF7/TamR cells was examined for the expression of genes associated with these processes using the RT^2^ Profiler PCR Array via qRT-PCR. With a threshold value of 2-fold expression change and a statistical significance of *P* < 0.05, the volcano plot shows the top 7-upregulated and 1-downregulated genes affected by the combined treatment in comparison to the control cells (Fig. 5a). Amongst them, *GADD45A* expression was upregulated in both the apoptosis and cell cycle arrays, which is consistent with previous studies showing that increased *GADD45A* expression leads to cell cycle arrest and apoptosis in a range of cell types, including breast cancer cells [17, 34]. Similarly, *BAX* and *FAS* were present in both the apoptosis and autophagy arrays, suggesting cross talk between apoptosis and autophagy induced by Cl-amidine combined with docetaxel treatment [35, 36]. We then validated the expression of these genes in each individual treatment and showed that the combined treatment also significantly increased the expression of *CDKN1A*, *GADD45A*, *FAS*, *BAG3*, *TNFRSF10B* rather than single agents alone (Fig. 5b). Again, either knockdown PAD2 or overexpression of miR-125b-5p had the similar effect as that of PAD2 inhibitor on the expression of these genes in MCF7/TamR cells treated with 0.1 μM docetaxel (Additional file 1: Figure S4). Importantly, gene ontology (GO) function analyses confirmed that these genes were enriched in p53 signaling pathway in cancers (Fig. 5c), which is consistent with a previous report [37], suggesting that the combined treatment may activate p53, which further regulates p53 target genes.

### Cl-amidine combined with docetaxel enhances p53 nuclear accumulation

**Fig. 6:**
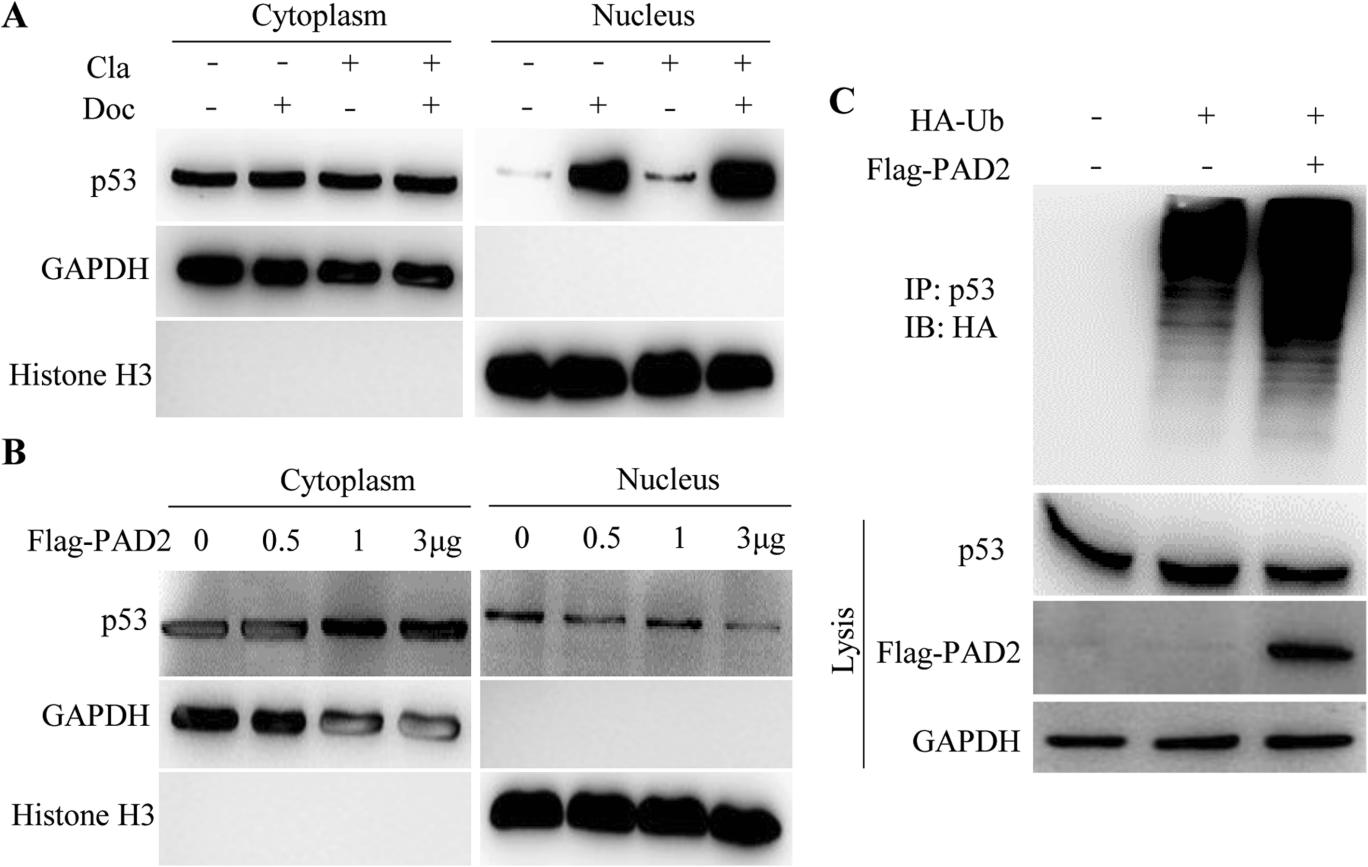
Inhibiting PAD2 combined with docetaxel treatment promoted nuclear accumulation of p53 in MCF7/TamR cells, and PAD2 facilitates p53 degradation by ubiquitination. **a** Cellular proteins after 0.1 μM docetaxel and 25 μM cl-amidine treatment were separated into cytoplasmic and nuclear pools by fractionation methods and examined by western blot with anti-p53 antibody. Cleanliness of fractionation was determined by probing with antibodies for Histone H3 (nuclear) and GAPDH (cytoplasmic) proteins. **b** HEK293 cells were transfected with Flag-tagged PAD2 followed by cellular fractionation analysis. Western blotting showing that cellular p53 expression was not affected by PAD2 overexpression, but nuclear p53 expression decreased. **c** HEK293 cells were transfected with HA-tagged ubiquitin (HA-Ub) and Flag-tagged PAD2, followed by immunoprecipitation assay by anti-p53 antibody. The immunoprecipitates were detected by anti-HA antibody. GAPDH served as loading control

The transcription activator p53 undergoes nuclear accumulation in response to several apoptotic stimuli, plays a central role in the induction of apoptosis, and thereby mediates cell cycle arrest in cancer cells [38, 39]. To test whether the combined treatment may also affect p53 nuclear accumulation in MCF7/TamR cells, we then separated nuclear and cytosolic fractions of the cells and subjected them to western blotting. We found that the cytosolic fraction of p53 was not affected by either individual drug or the combined treatment, compared to the controls (Fig. 6a). However, in agreement with the previous reports [38], docetaxel treatment dramatically induced nuclear p53 levels. Importantly, Cl-amidine activated nuclear p53 accumulation and this accumulation was further enhanced by the combined treatment (Fig. 6a). PAD2 knockdown or miR-125b-5p overexpression also promoted nuclear accumulation of p53 in MCF7/TamR cells treated with 0.1 μM docetaxel (Additional file 1: Figure S5). To further test the hypothesis that PAD2 regulates p53 nuclear accumulation, we overexpressed Flag-tagged PAD2 in HEK293 cells and then examined p53 expression. The results show that PAD2 overexpression does not affect the cytosolic expression of p53, but decreases nuclear p53 levels (Fig. 6b). Therefore, inhibiting PAD2 may play an essential role for this synergistic effect on p53 nuclear accumulation.

### PAD2 facilitates p53 degradation by ubiquitination

The molecular mechanism for how PAD2 regulates p53 is currently unknown. Given that p53 degradation, mediated by the E3 ubiquitin ligase Mdm2, is generally accepted as the major mechanism of p53 regulation [40, 41], we determined whether PAD2 may influence p53 ubiquitination. In that case, inhibiting PAD2 would be expected to prevent p53 degradation, which could explain the greatly promoted effect of Cl-amidine on p53 stability as observed in Fig. 6a. To test this hypothesis, we performed a ubiquitination assay (Fig. 6c). HA-tagged ubiquitin (HA-Ub) with both the presence and absence of Flag-tagged PAD2 were transfected into 293 cells and p53 was immunoprecipitated with an anti-p53 antibody. The ubiquitination status of p53 was determined by western blotting using an anti-HA antibody. Expression of PAD2 dramatically increased the ubiquitination of p53, suggesting that PAD2 facilitates p53 degradation by ubiquitination.

### Cl-amidine combined with docetaxel synergistically decreases the activation of Akt/mTOR signaling

**Fig. 7:**
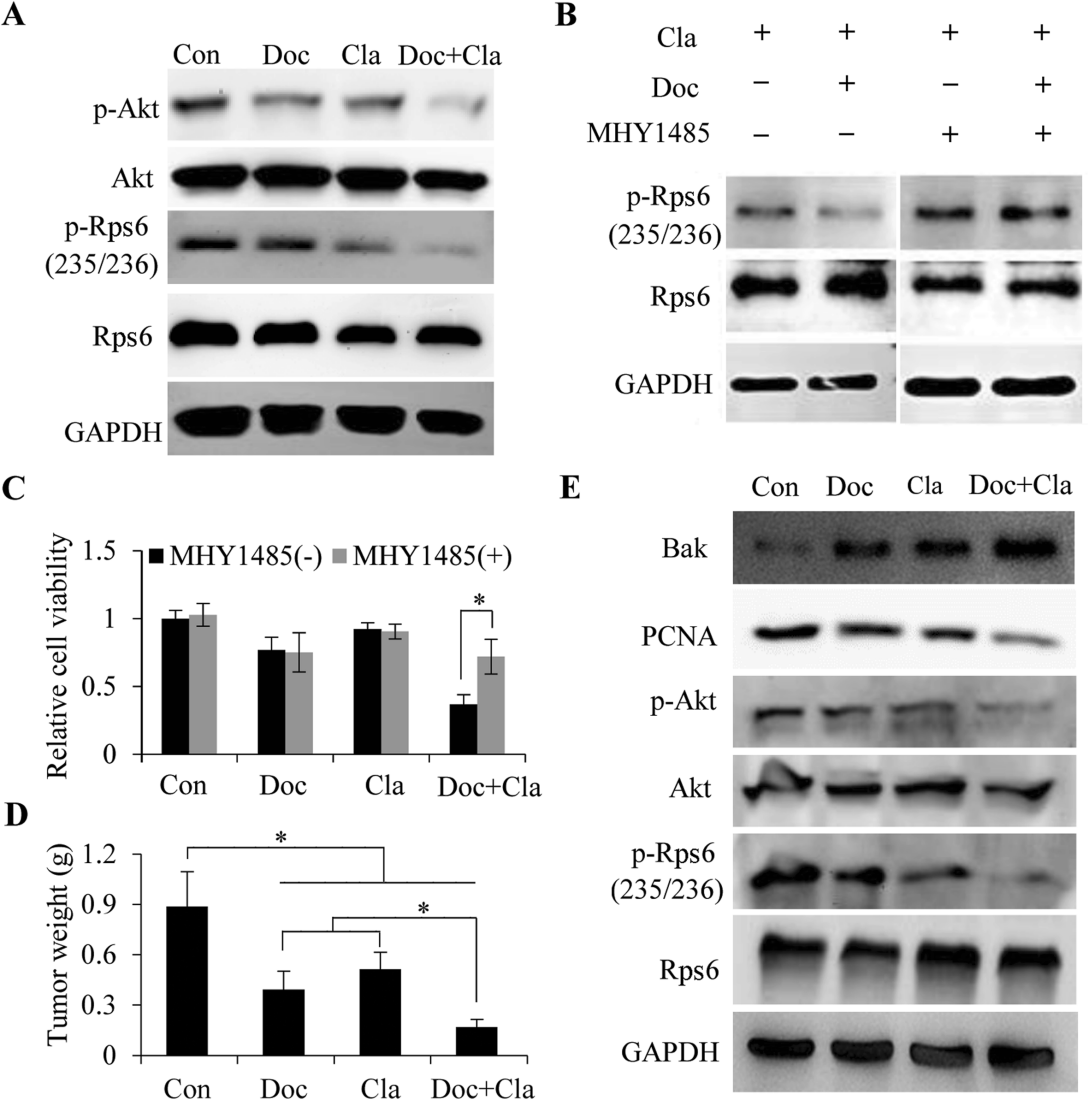
Cl-amidine combined with docetaxel synergistically decreases the activation of Akt/mTOR signaling. **a** Western blot analysis showing that the levels of p-Akt and p-Rps6 were decreased in MCF7/TamR cells treated with either docetaxel or cl-amidine, whereas a combination of docetaxel and cl-amidine nearly completely inhibited Akt and Rps6 phosphoryaltion. Total Akt and Rps6 proteins were not affected. GAPDH served as loading control. **b** MCF7/TamR cells were treated with 10 μM MHY1485, followed by cl-amidine and docetaxel treatment. Western blot analysis showing that pretreatment of cells with MHY1485 fully abolished the inhibitory effect of cl-amidine combined with docetaxel on Rps6 activation. GAPDH served as loading control. **c** MCF7/TamR cells were treated with 10 μM MHY1485, followed by cl-amidine and docetaxel treatment. CCK8 assay showing passively activating mTOR by MHY1485 reversed the inhibiting effect on viability of MCF7/TamR cells caused by cl-amidine and docetaxel combination treatment. (*P < 0.05). **d** MCF7/TamR cells were inoculated into the nude mice. Two weeks later, the mice were randomly divided into 4 groups and received PBS control, docetaxel, cl-amidine, or the combination injection (*n* = 6/group). The tumors were removed and the tumor weight was then recorded and plotted. *P < 0.05. **e** Western blot analysis of the mouse xenograft tumors with the antibodies against Bak, PCNA, p-Akt, Akt, p-Rps6, Rps6, and GAPDH

Accumulating studies have shown that p53 inhibits the mammalian target of rapamycin (mTOR) signaling pathway in response to cellular stresses [42, 43]. Wang et al. showed that inhibition of PAD4, another PAD family member, with inhibitor YW3–56, activates a cohort of p53 target genes, which in turn inhibits the mTORC1 signaling pathway, thereby perturbing autophagy and inhibiting cancerous cell growth [44]. Hence, we sought to determine whether Cl-amidine and docetaxel treatment also suppress Akt/mTOR signaling. To do this, we first showed that the levels of phosphorylated Akt and Rps6 were decreased in MCF7/TamR cells treated with either docetaxel or Cl-amidine, whereas a combination of docetaxel and Cl-amidine nearly completely inhibited Akt and Rps6 phosphorylation (Fig. 7a). PAD2 knockdown or miR-125b-5p overexpression also further decreased the levels of phosphorylated Akt and Rps6 phosphorylation in MCF7/TamR cells treated with 0.1 μM docetaxel (Additional file 1: Figure S6). Next, we treated MCF7/TamR cells with 10 μM MHY1485, a small-molecular mTOR activator, followed by Cl-amidine and docetaxel treatment. The results showed that pretreatment of cells with MHY1485 fully abolished the inhibitory effect of Cl-amidine combined with docetaxel on Rps6 activation (Fig. 7b). Furthermore, pretreatment of PAD2 knockdown or miR-125b-5p overexpression MCF7/TamR cells with MHY1485 also abolished the inhibitory effect of docetaxel on Rps6 activation (Additional file 1: Figure S7). Additionally, passively activating mTOR by MHY1485 also reversed the inhibiting effect on viability of MCF7/TamR cells caused by Cl-amidine and docetaxel treatment (Fig. 7c), as well as PAD2 knockdown or miR-125b-5p overexpression MCF7/TamR cells (Additional file 1: Figure S8). Furthermore, we tested the synergy of the PAD2 inhibitor and docetaxel in tumor growth in vivo (*n* = 6). We found that after injection of a mix of Cl-amidine and docetaxel, the tumor weight significantly decreased compared to that of each single treatment (Fig. 7d). As expected, the combined treatment also upregulated the expression of pro-apoptotic protein Bak and decreased the expression of cell proliferation marker PCNA in tumor tissues (Fig. 7e), indicating an additive effect of the two inhibitors. Correspondingly, the activation of Akt and Rps6 was strikingly inhibited after the combined treatment in the mouse xenograft tumors (Fig. 7e). Together, these results suggested that Cl-amidine and docetaxel may target upstream regulators for mTOR signaling, which help inhibit MCF7/TamR tumors.

## Discussion

Tamoxifen resistance in breast cancer therapy presents a huge clinical challenge [3, 4]. More detailed molecular mechanisms relevant to tamoxifen resistance and new therapy regimens benefit the breast cancer patients [45]. In our study, we first demonstrate that PAD2 is required for tamoxifen resistance in breast cancers and may represent a novel therapeutic target for tamoxifen resistance in breast cancers. Importantly, our study showing that PAD2 inhibition with Cl-amidine can partially restore the sensitivity of TamR/MCF7 cells to tamoxifen suggests that Cl-amidine may represent a new candidate for breast cancer therapy. Previous studies have shown that Cl-amidine causes cancer cell growth inhibition at 150–200 μM concentration in several breast cancer cell lines, while it does not affect the growth of the non-tumorigenic cells and was well tolerated by mice [17, 30, 46, 47]. Moreover, Cl-amidine combination with doxorubicin or the HDAC inhibitor SAHA can have synergistic cytotoxic effects on cells [31, 46–48]. In agreement with these reports, our study shows that combination treatment with 50 μM Cl-amidine and 5 μM tamoxifen exhibit a much stronger antiproliferative effect as compared to all individual treatments, though this inhibitory effect was not complete. To achieve better results, we applied a combination of 25 μM Cl-amidine and 0.1 μM docetaxel. This combined treatment nearly completely inhibited MCF7/TamR cells. To our knowledge, this is the lowest concentration of Cl-amidine (6–8-fold lower than the regular dosage used in treating cancer cells) reported so far to have such a significant inhibitory effect on cancer cells. In addition, this drug regimen also decreased docetaxel dosage from 80 μM when used alone to 0.1 μM when used in combination. The synergy of the PAD2 inhibitor and docetaxel was also confirmed in tumor growth in vivo. High-dose docetaxel therapy was found to be associated with safety issues among clinical trial patients and effective strategies to decrease the dosage are urgently needed [5, 10]. Therefore, our drug regimen may provide a better therapeutic strategy to reduce the dose of docetaxel used for those patients with tamoxifen-resistant breast cancer in the future.

**Fig. 8:**
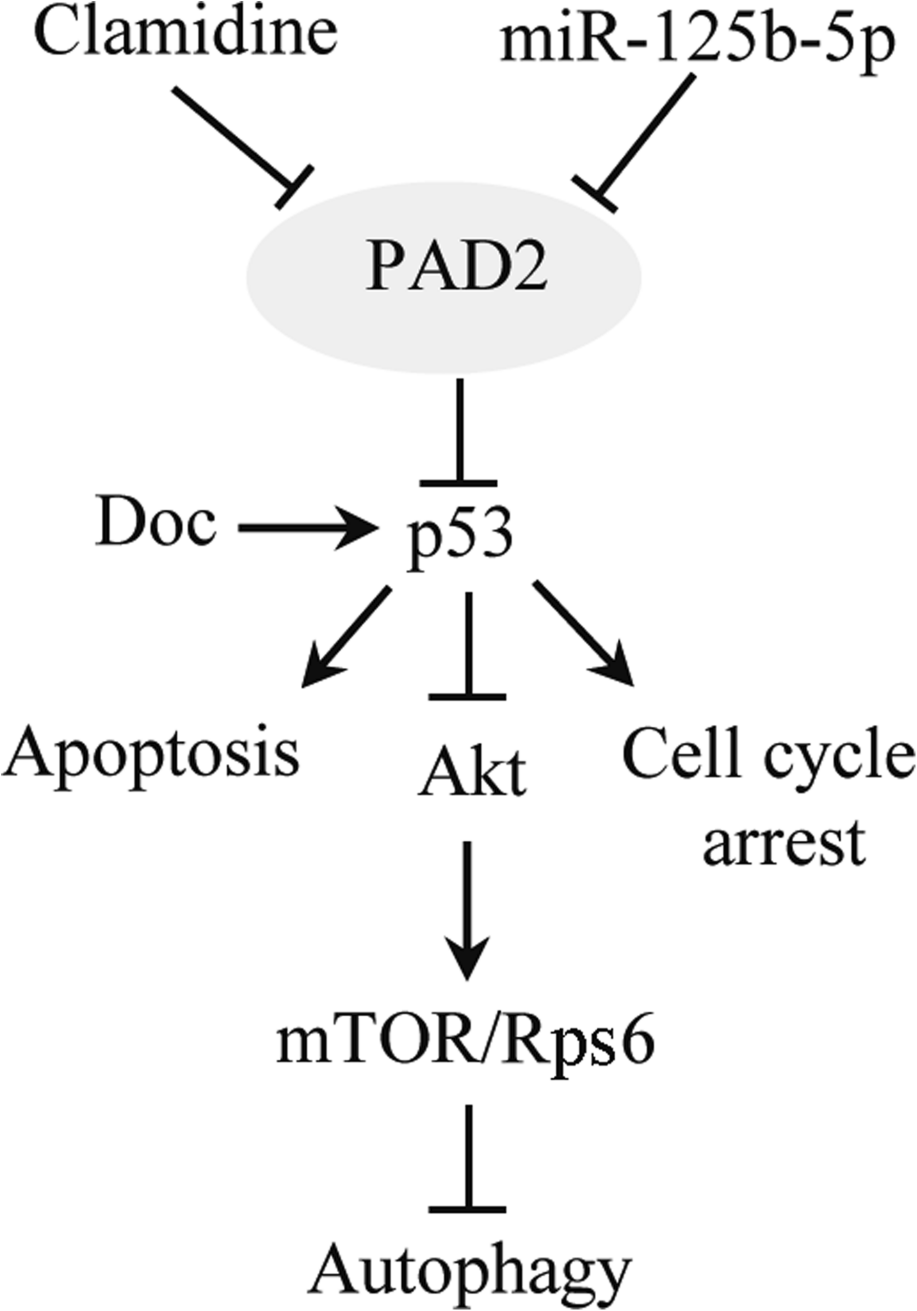
Schematic of the synergistic effects of cl-amidine and docetaxel on p53/Akt/mTOR signaling in regulation of genes associated with cell apoptosis, cell cycle arrest and autophagy. Inhibiting PAD2 reverses p53 degradation, leading to the enhanced p53 nuclear accumulation with docetaxel co-treatment, thus accelerating cell apoptosis and cell cycle arrest. The combination treatment also promotes the autophagy processes by synergistically inhibiting Akt/mTOR signing, leading to the enhanced proliferation inhibition

p53 plays a pivotal role in controlling cell-cycle progression and apoptosis [40]. It has been shown that PAD2 is involved in regulating the expression of both cell cycle and tumor promoting genes [17] and inhibiting PAD2 with Cl-amidine effectively upregulates several p53-regulated genes, leading to an increase in apoptosis and cell cycle arrest [47, 48]. Meanwhile, docetaxel can upregulate p53 expression, and treatment of breast cancer cells with docetaxel induces a sustained arrest in mitosis with the consequent inhibition of transcription and higher p53 accumulation followed by apoptosis [32]. In agreement with these findings, our study showed synergism between Cl-amidine and docetaxel in enhancing the activation of p53, and that the combination treatment synergistically induced apoptosis and cell cycle arrest in the G2/M phase (Fig. 8). Regarding the molecular mechanism of the additive effect, we showed that PAD2 promotes p53 ubiquitination and its subsequent degradation. Inhibiting PAD2 reversed p53 degradation, leading to enhanced p53 nuclear accumulation with docetaxel co-treatment. Of note, the combination treatment also induced autophagy in MCF7/TamR cells. Autophagy exhibits either a protumorigenic or antitumorigenic function, depending on the cell type, developmental stage of cancer and stimulator [49]. Wang and colleagues have shown that PAD inhibitors activate p53, which in turn inhibits the mTOR signaling pathway, and induces autophagy and cancer growth inhibition [23, 25, 45]. Docetaxel has also been reported to induce breast cancer cell autophagy [42]. Given that both Cl-amidine and docetaxel can activate p53, the combination treatment in our study may accelerate the autophagy processes by synergistically inhibiting Akt/mTOR signing, thus leading to the enhanced proliferation inhibition.

In our study, we only used cell line-derived xenograft mouse models and could not use patient-derived xenografts, since it is difficult to obtain patient-derived ER-positive xenografts. We will test our hypothesis in patient-derived ER-positive xenografts in our future studies once the samples are available. We hope that with this model, we will provide more predictive values for clinical outcomes. Additionally, to our knowledge, tamoxifen is still the first-line endocrine therapy for premenopausal, ER positive metastatic breast cancer in China, while fulvestrant as ER antagonis, is usually preferred in postmenopausal women with advanced breast cancer [50]. It is currently unknown whether PAD2 is also involved in fulvestrant resistance breast cancer or not. Clearly, the role of PAD2 in fulvestrant resistance breast cancer still needs to be determined.

## Conclusions

We provided here an important new line of evidence demonstrating that PAD2 was highly expressed in tamoxifen-resistant breast cancer, raising a possibility that upregulated PAD2 may be involved in tamoxifen-resistance in breast cancer. Treatment with PADs inhibitor can partially resensitize TamR/MCF7 cells to tamoxifen. Most importantly, we demonstrated a synergistic and effective drug regimen of PADs inhibitor and docetaxel (with lower concentrations of both drugs) on tamoxifen-resistant breast cancer cells. Such combination therapy may be a novel potential therapeutic approach for patients with this form of breast cancer.

## Supplementary information

**Additional file 1: Figure S1.** Flow cytometric analysis of PAD2 knockdown (**a**) or miR-125b-5p overexpression (**b**) accelerated apoptosis of the MCF7/TamR cells treated with 0.1 μM docetaxel. Relative apoptosis ratio is quantified on the right. shCon: shRNA control MCF7/TamR cells; shPAD2: PAD2 knockdown cells; EV con: Empty vector pQXCIP overexpression MCF7/TamR cells; miR-125b-5p: miR-125b-5p overexpression; Doc: docetaxel; PBS was used as a control.

**Additional file 2: Figure S2.** Flow cytometric analysis of PAD2 knockdown (**a**) or miR-125b-5p overexpression (**b**) induced a stronger cell cycle arrest in the G2/M phase of the MCF7/TamR cells treated with 0.1 μM docetaxel. The relative quantification was shown below the representative images. shCon: shRNA control MCF7/TamR cells; shPAD2: PAD2 knockdown cells; EV con: Empty vector pQXCIP overexpression MCF7/TamR cells; miR-125b-5p: miR-125b-5p overexpression; Doc: docetaxel; PBS was used as a control. (*P < 0.05).

**Additional file 3: Figure S3.** Western blot analysis of the PAD2 knockdown (**a**) or miR-125b-5p overexpression (**b**) accumulated LC3B protein expression in the MCF7/TamR cells treated with 0.1 μM docetaxel. shCon: shRNA control MCF7/TamR cells; shPAD2: PAD2 knockdown cells; EV con: Empty vector pQXCIP overexpression MCF7/TamR cells; miR-125b-5p: miR-125b-5p overexpression; Doc: docetaxel; PBS was used as a control. GAPDH served as loading control.

**Additional file 4: Figure S4.** qRT-PCR analysis showing that PAD2 knockdown (**a**) or miR-125b-5p overexpression (**b**) significantly increased the expression of CDKN1A, GADD45A, FAS, BAG3, TNFRSF10B in the MCF7/TamR cells treated with 0.1 μM docetaxel. shCon: shRNA control MCF7/TamR cells; shPAD2: PAD2 knockdown cells; EV con: Empty vector pQXCIP overexpression MCF7/TamR cells; miR-125b-5p: miR-125b-5p overexpression; Doc: docetaxel; PBS was used as a control. Gene expression normalized to GAPDH. (*P < 0.05).

**Additional file 5. Figure S5.** Western blot analysis of the PAD2 knockdown (**a**) or miR-125b-5p overexpression (**b**) promoted nuclear accumulation of p53 in MCF7/TamR cells treated with 0.1 μM docetaxel. Cellular proteins after 0.1 μM docetaxel treatment were separated into cytoplasmic and nuclear pools by fractionation methods and examined by western blot with anti-p53 antibody. Cleanliness of fractionation was determined by probing with antibodies for Pol II (nuclear) and GAPDH (cytoplasmic) proteins. shCon: shRNA control MCF7/TamR cells; shPAD2: PAD2 knockdown cells; EV con: Empty vector pQXCIP overexpression MCF7/TamR cells; miR-125b-5p: miR-125b-5p overexpression; Doc: docetaxel; PBS was used as a control.

**Additional file 6. Figure S6.** Western blot analysis of the PAD2 knockdown (**a**) or miR-125b-5p overexpression (**b**) further decreased the levels of phosphorylated Akt and Rps6 phosphorylation in MCF7/TamR cells treated with 0.1 μM docetaxel. GAPDH served as loading control. shCon: shRNA control MCF7/TamR cells; shPAD2: PAD2 knockdown cells; EV con: Empty vector pQXCIP overexpression MCF7/TamR cells; miR-125b-5p: miR-125b-5p overexpression; Doc: docetaxel; PBS was used as a control.

**Additional file 7. Figure S7.** Western blot analysis showing that pretreatment of PAD2 knockdown (**a**) or miR-125b-5p overexpression (**b**) MCF7/TamR cells with 10 μM MHY1485 abolished the inhibitory effect of docetaxel on Rps6 activation. GAPDH served as loading control; shPAD2: PAD2 knockdown MCF7/TamR cells; miR-125b-5p: miR-125b-5p overexpression MCF7/TamR cells; Doc: docetaxel; PBS was used as a control.

**Additional file 8. Figure S8.** CCK8 assay showing passively activating mTOR by MHY1485 reversed the inhibiting effect of docetaxel on viability of PAD2 knockdown (**a**) or miR-125b-5p overexpression (**b**) MCF7/TamR cells. shCon: shRNA control MCF7/TamR cells; shPAD2: PAD2 knockdown cells; EV con: Empty vector pQXCIP overexpression MCF7/TamR cells; miR-125b-5p: miR-125b-5p overexpression cells; Doc: docetaxel; PBS was used as a control. (*P < 0.05).

**Additional file 9: Table S1.** qRT-PCR primer sequences used in the study.

## Data Availability

All data generated or analyzed during this study are included in this published article and its supplementary information files.

## Abbreviations

CCK-8: Cell counting kit-8
DMEM: Dulbecco’s Modified Eagle Medium
ER: Estrogen receptor
FBS: Fetal bovine serum
FITC: Fluorescein isothiocyanate
LC3B: Microtubule-associated protein light chain 3
mTOR: Mammalian target of rapamycin
OD: Optical density
PAD2: Peptidylarginine deiminase 2
PBS: Phosphate-buffered saline
PI: Propidium iodide
RIPA: Radioimmunoprecipitation assay
shRNA: short hairpin RNA
TamR: Tamoxifen-resistant
TamS: Tamoxifen-sensitive
TUNEL: Terminal deoxynucleotidyltransferase mediated dUTP-biotin nick end labeling
WT: Wild-type
