# Supplementary material for: Inhibiting PAD2 enhances the anti-tumor effect of docetaxel in tamoxifen-resistant breast cancer cells
Source: J Exp Clin Cancer Res. 2019 Oct 10;38:414. doi: 10.1186/s13046-019-1404-8 (PMC6785896; doi:10.1186/s13046-019-1404-8)
Supplement: Supplementary file 1 — Additional file 1: Figure S1. Flow cytometric analysis of PAD2 knockdown (a) or miR-125b-5p overexpression (b) accelerated apoptosis of the MCF7/TamR cells treated with 0.1 μM docetaxel. Relative apoptosis ratio is quantified on the right. shCon: shRNA control MCF7/TamR cells; shPAD2: PAD2 knockdown cells; EV con: Empty vector pQXCIP overexpression MCF7/TamR cells; miR-125b-5p: miR-125b-5p overexpression; Doc: docetaxel; PBS was used as a control. [file 13046_2019_1404_MOESM1_ESM.docx]

**Additional file 1**

**Figure S1.** Flow cytometric analysis of PAD2 knockdown (**a**) or miR-125b-5p overexpression (**b**) accelerated apoptosis of the MCF7/TamR cells treated with 0.1 μM docetaxel. Relative apoptosis ratio is quantified on the right. shCon: shRNA control MCF7/TamR cells; shPAD2: PAD2 knockdown cells; EV con: Empty vector pQXCIP overexpression MCF7/TamR cells; miR-125b-5p: miR-125b-5p overexpression; Doc: docetaxel; PBS was used as a control.
