# Supplementary material for: Inhibiting PAD2 enhances the anti-tumor effect of docetaxel in tamoxifen-resistant breast cancer cells
Source: J Exp Clin Cancer Res. 2019 Oct 10;38:414. doi: 10.1186/s13046-019-1404-8 (PMC6785896; doi:10.1186/s13046-019-1404-8)
Supplement: Supplementary file 5 — Additional file 5. Figure S5. Western blot analysis of the PAD2 knockdown (a) or miR-125b-5p overexpression (b) promoted nuclear accumulation of p53 in MCF7/TamR cells treated with 0.1 μM docetaxel. Cellular proteins after 0.1 μM docetaxel treatment were separated into cytoplasmic and nuclear pools by fractionation methods and examined by western blot with anti-p53 antibody. Cleanliness of fractionation was determined by probing with antibodies for Pol II (nuclear) and GAPDH (cytoplasmic) proteins. shCon: shRNA control MCF7/TamR cells; shPAD2: PAD2 knockdown cells; EV con: Empty vector pQXCIP overexpression MCF7/TamR cells; miR-125b-5p: miR-125b-5p overexpression; Doc: docetaxel; PBS was used as a control. [file 13046_2019_1404_MOESM5_ESM.docx]

**Additional file 5**

**Figure S5.** Western blot analysis of the PAD2 knockdown (**a**) or miR-125b-5p overexpression (**b**) promoted nuclear accumulation of p53 in MCF7/TamR cells treated with 0.1 μM docetaxel. Cellular proteins after 0.1 μM docetaxel treatment were separated into cytoplasmic and nuclear pools by fractionation methods and examined by western blot with anti-p53 antibody. Cleanliness of fractionation was determined by probing with antibodies for Pol II (nuclear) and GAPDH (cytoplasmic) proteins. shCon: shRNA control MCF7/TamR cells; shPAD2: PAD2 knockdown cells; EV con: Empty vector pQXCIP overexpression MCF7/TamR cells; miR-125b-5p: miR-125b-5p overexpression; Doc: docetaxel; PBS was used as a control.
