# Supplementary material for: Inhibiting PAD2 enhances the anti-tumor effect of docetaxel in tamoxifen-resistant breast cancer cells
Source: J Exp Clin Cancer Res. 2019 Oct 10;38:414. doi: 10.1186/s13046-019-1404-8 (PMC6785896; doi:10.1186/s13046-019-1404-8)
Supplement: Supplementary file 7 — Additional file 7. Figure S7. Western blot analysis showing that pretreatment of PAD2 knockdown (a) or miR-125b-5p overexpression (b) MCF7/TamR cells with 10 μM MHY1485 abolished the inhibitory effect of docetaxel on Rps6 activation. GAPDH served as loading control; shPAD2: PAD2 knockdown MCF7/TamR cells; miR-125b-5p: miR-125b-5p overexpression MCF7/TamR cells; Doc: docetaxel; PBS was used as a control. [file 13046_2019_1404_MOESM7_ESM.docx]

**Additional file 7**

**Figure S7.** Western blot analysis showing that pretreatment of PAD2 knockdown (**a**) or miR-125b-5p overexpression (**b**) MCF7/TamR cells with 10 μM MHY1485 abolished the inhibitory effect of docetaxel on Rps6 activation. GAPDH served as loading control; shPAD2: PAD2 knockdown MCF7/TamR cells; miR-125b-5p: miR-125b-5p overexpression MCF7/TamR cells; Doc: docetaxel; PBS was used as a control.
