# Supplementary material for: Inhibiting PAD2 enhances the anti-tumor effect of docetaxel in tamoxifen-resistant breast cancer cells
Source: J Exp Clin Cancer Res. 2019 Oct 10;38:414. doi: 10.1186/s13046-019-1404-8 (PMC6785896; doi:10.1186/s13046-019-1404-8)
Supplement: Supplementary file 9 — Additional file 9: Table S1. qRT-PCR primer sequences used in the study. [file 13046_2019_1404_MOESM9_ESM.docx]

**Additional file 9**

**Table S1. qRT-PCR primer sequences used in the study**

| **Gene** | **Primer** | **Sequence (5′-3′)** |
| --- | --- | --- |
| PAD1: | Forward | GAG TGA TGG ACA CTC ATG GC |
|  | Reverse | CAG ATG GTC AGC TTG CAG TT |
| PAD2 | Forward | TCT CAG GCC TGG TCT CCA T |
|  | Reverse | AAG ATG GGA GTC AGG GGA AT |
| PAD3 | Forward | AGC AAT GAC CTC AAC GAC AG |
|  | Reverse | TGA GGT AGA GCA CCG CAT AG |
| PAD4 | Forward | TCA CCT ACC ACA TCA GGC AT |
|  | Reverse | CAT GTT CCA CCA CTT GAA GG |
| miR-125b-5p | Forward | TCC CTG AGA CCC TAA CTT GTG A |
|  | Reverse | CAG TGC GTG TCG TGG AGT |
| U6 | Forward | CTC GCT TCG GCA GCA CAT ATA CT |
|  | Reverse | ACG CTT CAC GAA TTT GCG TGT C |
| CDKN1A | Forward | TGT CCG TCA GAA CCC ATG C |
|  | Reverse | AAA GTC GAA GTT CCA TCG CTC |
| GADD45A | Forward | CTG CGA GAA CGA CAT CAA |
|  | Reverse | CCT TCC ATT GAG ATG AAT GTG |
| TNFRSF10B | Forward | GCA GCC GTA GTC TTG ATT |
|  | Reverse | CGA TCT CAT TGA GGA CAT TG |
| FAS | Forward | TCG TAA TTG GCA TCA ACT TC |
|  | Reverse | CCT TGA GGA TGA TAG TCT GAA |
| BAG3 | Forward | AAG GCA AGA AGA CTG ACA A |
|  | Reverse | ATA GAC CTG GAC TTG ACC T |
| BAX | Forward | CAC TGA AGC GAC TGA TGT |
|  | Reverse | CTT CTT CCA GAT GGT GAG T |
| MDM2 | Forward | GAA TCA TCG GAC TCA GGT ACA TC |
|  | Reverse | TCT GTC TCA CTA ATT GCT CTC CT |
| AURKB | Forward | CAG TGG GAC ACC CGA CAT C |
|  | Reverse | GTA CAC GTT TCC AAA CTT GCC |
| GAPDH | Forward | ACC CAT CAC CAT CTT CCA GGA G |
|  | Reverse | GAA GGG GCG GAG ATG ATG AC |
